# Quantitative Electroencephalography as a Biomarker for Cognitive Dysfunction in Parkinson’s Disease

**DOI:** 10.3389/fnagi.2021.804991

**Published:** 2022-01-03

**Authors:** Kevin Novak, Bruce A. Chase, Jaishree Narayanan, Premananda Indic, Katerina Markopoulou

**Affiliations:** ^1^Department of Neurology, NorthShore University HealthSystem, Evanston, IL, United States; ^2^Department of Neurology, Pritzker School of Medicine, University of Chicago, Chicago, IL, United States; ^3^Department of Health Information Technology, Clinical Analytics, NorthShore University HealthSystem, Evanston, IL, United States; ^4^Department of Electrical Engineering, The University of Texas at Tyler, Tyler, TX, United States

**Keywords:** quantitative electroencephalography (QEEG), biomarker, Parkinson’s disease, cognitive dysfunction, wavelet-based time-transform algorithm

## Abstract

**Background:** Quantitative electroencephalography (qEEG) has been suggested as a biomarker for cognitive decline in Parkinson’s disease (PD).

**Objective:** Determine if applying a wavelet-based qEEG algorithm to 21-electrode, resting-state EEG recordings obtained in a routine clinical setting has utility for predicting cognitive impairment in PD.

**Methods:** PD subjects, evaluated by disease stage and motor score, were compared to healthy controls (*N* = 20 each). PD subjects with normal (PDN, MoCA 26–30, *N* = 6) and impaired (PDD, MoCA ≤ 25, *N* = 14) cognition were compared. The wavelet-transform based time-frequency algorithm assessed the instantaneous predominant frequency (IPF) at 60 ms intervals throughout entire recordings. We then determined the relative time spent by the IPF in the four standard EEG frequency bands (RTF) at each scalp location. The resting occipital rhythm (ROR) was assessed using standard power spectral analysis.

**Results:** Comparing PD subjects to healthy controls, mean values are decreased for ROR and RTF-Beta, greater for RTF-Theta and similar for RTF-Delta and RTF-Alpha. In logistic regression models, arithmetic combinations of RTF values [e.g., (RTF-Alpha) + (RTF-Beta)/(RTF-Delta + RTF-Theta)] and RTF-Alpha values at occipital or parietal locations are most able to discriminate between PD and controls. A principal component (PC) from principal component analysis (PCA) using RTF-band values in all subjects is associated with PD status (*p* = 0.004, β = 0.31, AUC = 0.780). Its loadings show positive contribution from RTF-Theta at all scalp locations, and negative contributions from RTF-Beta at occipital, parietal, central, and temporal locations. Compared to cognitively normal PD subjects, cognitively impaired PD subjects have lower median RTF-Alpha and RTF-Beta values, greater RTF-Theta values and similar RTF-Delta values. A PC from PCA using RTF-band values in PD subjects is associated with cognitive status (*p* = 0.002, β = 0.922, AUC = 0.89). Its loadings show positive contributions from RTF-Theta at all scalp locations, negative contributions from RTF-Beta at central locations, and negative contributions from RTF-Delta at central, frontal and temporal locations. Age, disease duration and/or sex are not significant covariates. No PC was associated with motor score or disease stage.

**Significance:** Analyzing standard EEG recordings obtained in a community practice setting using a wavelet-based qEEG algorithm shows promise as a PD biomarker and for predicting cognitive impairment in PD.

## Introduction

Parkinson’s disease (PD) is characterized by the presence of cardinal motor symptoms including resting tremor, rigidity, bradykinesia and postural instability. Non-motor symptoms including cognitive impairment, anxiety, depression, REM-sleep-behavior disorder, olfactory dysfunction, autonomic dysfunction, orthostatic hypotension, urinary incontinence and constipation are common and often precede the onset of motor symptoms. Cognitive impairment becomes more prevalent as the disease progresses and dementia often develops (Aarsland and Kurz, 2010). The prevalence of dementia in PD may be as high as 75% for disease duration greater than 10 years (Hely et al., 2008). The ability to predict the onset or likelihood of cognitive impairment in PD is important, as impaired cognition is associated with increased morbidity and mortality, decreased quality of life, and increased caregiver burden.

Biomarkers of cognitive dysfunction in PD have been developed (Shi et al., 2010) based on neuroimaging (Poston and Eidelberg, 2009; Arnaldi et al., 2017), genetics (Liu et al., 2017), blood (Lawton et al., 2020) and CSF (Mollenhauer et al., 2017). Over the last decade, analyses of non-stationary EEG data, data where the statistical characteristics change with time, using quantitative electroencephalography (qEEG) methods have been investigated for their ability to distinguish the parkinsonian state and/or predict dementia in PD. Different measures and EEG characteristics have been utilized for this purpose: Fonseca et al. (2009) observed an increase in absolute and relative posterior-delta amplitude in 32 PD patients compared to 26 normal controls; Morita et al. (2011) found an association between a decrease in the spectral ratio of fast-to-slow frequency bands [(Alpha + Beta)/(Theta + Delta)] and a decline in Mini-Mental State Examination (MMSE) scores; Caviness et al. (2015, 2016) reported that an increase in delta-band power was associated with longitudinal changes in neuropsychological testing and distinguished PD patients from controls; and Klassen et al. (2011) found that background frequency and relative power in the theta band were potential predictors of dementia in PD in a cohort of 138 PD subjects, 21 of which had dementia at baseline.

Most of the above-mentioned studies included patients who were undergoing dopaminergic therapy. To address the contributions of potential treatment effects as well as disease–stage, Arnaldi et al. (2017) analyzed EEGs in a cohort of 57 drug-naïve *de novo* PD patients using qEEG and ^123^I-FP-CIT-SPECT and determined that a mean posterior qEEG frequency of less than 8.3 Hz was a good predictor of cognitive decline in that cohort. Chaturvedi et al. (2017) analyzed high-resolution 256–channel EEG recordings of PD patients and found that theta-power in the left temporal region and the alpha/theta ratio in the central and left regions were able to distinguish PD patients from healthy controls, suggesting a role for regional EEG differences in the disease state. Geraedts et al. (2018) showed that decreased posterior dominant frequency and increased theta power correlate with cognitive impairment in PD. Massa et al. (2020) showed that the alpha/theta ratio was significantly lower in early stage Lewy body disease and in PD without cognitive impairment in comparison to healthy controls and Alzheimer’s-disease-associated mild cognitive impairment (MCI-AD). Using qEEG, Schumacher et al. (2020) compared mild cognitive impairment associated with Lewy bodies (MCI-LB) with MCI-AD and reported increased pre-alpha, decreased beta power and slower dominant frequency in MCI-LB, suggesting that early EEG slowing is a specific feature of MCI-LB. Taken together, these studies demonstrate that EEG and qEEG parameters can not only distinguish PD patients from healthy controls, but also serve as potential biomarkers of cognitive impairment in PD and LB-associated diseases. In addition, they point to the association of regional EEG differences with the disease state and degree of cognitive impairment.

An open question is how to best harness the discriminatory ability of qEEG for analyzing non-stationary EEG data for the purpose of distinguishing healthy controls from PD subjects, and cognitively normal from cognitively impaired PD subjects, in clinical settings where 21-electrode resting-state EEG data is routinely obtained. In this report, we evaluate whether a wavelet-based algorithm has utility for this purpose. Since EEG data can contain multiple independent spectral components that can vary rapidly in their frequency, we hypothesized that PD subjects differ from healthy controls, and cognitively normal PD subjects differ from cognitively impaired PD subjects, in how these spectral components change in frequency. To capture the rapid changes in frequency, we applied a wavelet-based algorithm previously developed by two of us (Indic and Narayanan, 2011) to derive the instantaneous frequency of the EEG signal at multiple time scales. We then determined the instantaneous predominant frequency (IPF, F0) at 60-msec intervals throughout the entire EEG recording at each scalp location and characterized the relative time spent by the IPF in each of the four standard (alpha, beta, delta, theta) EEG frequency bands (RTF). We analyzed these data using logistic regression and principal components analysis (PCA) to assess whether the RTF values in one or more EEG frequency bands (RTF-band) or arithmetic combinations of these values [e.g., the ratio of Alpha + Beta to Delta + Theta, after Morita et al. (2011)] can be used to distinguish PD from healthy control subjects, and within the PD cohort, subjects with normal cognition from subjects with cognitive impairment.

## Materials and Methods

This retrospective review of patient records and EEG data study was approved by the NorthShore institutional review board.

### Study Participants

**FIGURE 1:**
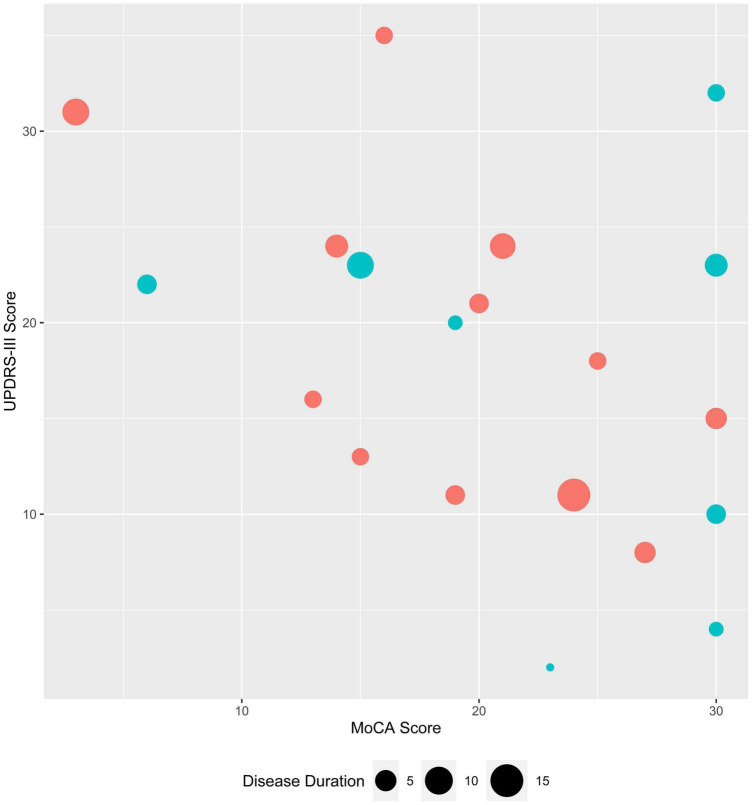
Clinical characteristics of study subjects with Parkinson’s disease. MoCA and UPDRS-III scores are plotted for female (teal) and male (coral) PD subjects; symbol size corresponds to disease duration (range: 1–15 years),

Twenty PD patients were recruited from one author’s (KM) movement disorders clinic. These subjects were medicated with levodopa or dopamine agonists at the time of EEG recording. They remained on medications in consideration of Best Practice and to be consistent with the aim of evaluating whether qEEG analysis of routinely collected EEG data, which would be collected while patients remain on their medications, is productive for discriminating between the groups being studied. The twenty age and sex-matched healthy controls were subjects whose EEGs were performed at our health system following a syncopal episode. Healthy controls had no neurological diagnoses in their medical records and a normal EEG. The average duration of EEG recording was 40 min (range: 30–60 min). The Montreal Cognitive Assessment (MoCA), which is the recommended scale for capturing cognitive changes in PD (Chou et al., 2010), the Unified Parkinson’s Disease Rating Scale (UPDRS) part III (motor) (Fahn et al., 1987; Movement Disorder Society Task Force on Rating Scales for Parkinson’s Disease, 2003) and the Hoehn and Yahr (H&Y) disease-stage scale (Goetz et al., 2004) scores were obtained as part of the standard clinical evaluation of PD patients in our movement disorders clinic at the NorthShore University HealthSystem (Evanston, IL). Twelve males and eight females were included in each group with an average age of 72.4 (standard deviation (*SD*) 8.1, range 59–85) and 72.7 (*SD* 8.4, range 59–89) years, for PD and control groups, respectively. Clinical features of the PD patients are shown in Figure 1.

### Quantitative Electroencephalography Analysis

Natus Neuroworks^®^ (Middleton, WI) was used for EEG recording and review. Raw EEG data from each channel were exported to MATLAB (Version 2018. The Math Works Inc., MA) for the wavelet analysis. To capture the rapid variation in frequency in the non-stationary EEG signal, we used an algorithm previously developed by two of us (Indic and Narayanan, 2011) to derive the instantaneous frequencies seen in the EEG signal at multiple time scales. This algorithm uses a continuous wavelet transform, with the Morlet wavelet as the mother wavelet, to complete a time-frequency decomposition of the EEG signal and extract information about simultaneously existing frequencies in the EEG data. It can reliably extract frequency information even in the presence of artifacts, providing they do not have the same frequency content of the EEG (Indic and Narayanan, 2011).

The wavelet transform of the discrete EEG signal is obtained by the convolution of the scaled and translated version of a mother wavelet function with the signal. The convolution is performed N times, N being the length of the EEG signal, using a discrete Fourier transform and wavelet function at each of a set of predefined time scales. We used a dyadic representation of time scales with eight sub-octave scales per octave with a total of 80 scales. The wavelet transformation provides a scalogram—a visual representation plotting the absolute value of the continuous wavelet transform as a function of time and frequency, and so is useful for time-localization of short-duration of high-frequency events as well as frequency localization of low-frequency, longer duration events. At each instant of time, we estimated the peak value of the scalogram. The frequency corresponding to the peak at an instant of time provides the instantaneous predominant frequency (IPF, or F0). The output of the analysis for each EEG channel was the IPF over time, with calculation of the IPF performed every 60 ms. For each channel we calculated the probability of IPF being in one of the four, standard-EEG-frequency bands (delta, theta, alpha, or beta) over the entire EEG recording. We refer to this as the relative time of this frequency (RTF) in each band at that channel.

Analysis of the resting occipital rhythm (ROR) was performed with *Spike2* (Cambridge Electronic Design, Cambridge, United Kingdom) and Igor Pro software (Wave metrics, Lake Oswego, OR). Standard power-spectral-density analysis was performed over a short block of time while the subject was resting with their eyes closed, using a Fast Fourier Transform (FFT).

### Statistical Analyses

Since this is a pilot study aimed at evaluating whether the wavelet algorithm previously described by Indic and Narayanan (2011) can be applied to resting-state EEG data to identify features associated with disease (PD vs. healthy control) and cognitive status in PD subjects (normal vs. impaired cognition), there were no published data allowing us to estimate an effect size for the differences that would be seen in either of these groups. Therefore, we could not perform an *a priori* power analysis. We assessed 20 PD and 20 healthy controls; within the PD subjects, six were cognitively normal (PDN, MoCA 26-30) while 14 were cognitively impaired (PDD, MoCA ≤ 25). This sample size is comparable to that used in some studies that identified differences in these groups using other EEG analysis methods (see section “Introduction”). We report 95% confidence intervals (CIs) to allow for independent determination of the effect size and range of possible differences with which our data are consistent.

Differences in RTF-band values between subject groups (PD vs. healthy control, PDN vs. PDD) were initially evaluated using both parametric (two-tailed *t*-test (TT) to compare means (*M*) and nonparametric [median test (MT) to compare medians (*MD*), Mann-Whitney *U*-test (MWU) to compare distributions] statistics. Logistic regression models (Stata, vs. 16.1, StataCorp LLC, TX) were used to evaluate associations between subject groups and RTF-band values or, since specific spectral differences have been associated with cognition in previous studies (see Introduction), arithmetic combinations of RTF-band values [(RTF-Alpha + RTF-Beta), (RTF-Alpha + RTF-Beta – RTF-Theta), (RTF-Alpha – RTF-Theta), (RTF-Theta – RTF-Beta), and (RTF-Alpha + RTF-Beta)/(RTF-Delta + RTF-Theta)], by location, focusing on variables with relatively high and similar variance in PD and control subjects. Given the size of groups in our cohort, we sought to minimize overfitting (Peduzzi et al., 1996; Vittinghoff and McCulloch, 2007) in these exploratory models by considering the contributions of only one or two RTF-band variables at once, e.g., RTF-Theta at O1 and O2 or P3 and P4, and interpreted significant models (model *p* and RTF-band variable *p* < 0.05) as providing evidence of association only if they also passed a link test, a Pearson goodness-of-fit test, and had a McFadden’s adjusted *R*^2^ supportive of good model fit (0.2–0.4). The Aikake information criterion (AIC) was used to evaluate whether model fit was improved by including age, sex, and/or disease duration as covariates.

Since we captured four RTF-band values at each of 21 scalp locations in each study subject, we used principal components analysis (PCA), performed in Stata (vs. 16.1) and in *R*,^1^ on data from 15 scalp locations to reduce data dimensionality and reduce the likelihood of false discovery. PCA provides an unsupervised method to identify a low-dimensional set of uncorrelated variables that effectively summarize the data obtained from applying the wavelet transform algorithm to EEG data at each scalp location. The RTF-band data for a particular frequency band at different scalp locations tend to be correlated and consequently reflect redundant properties of the recorded brain activity. In the PCAs we performed, we used only RTF-band data, so each PCA used the variance of the RTF-band values obtained at each scalp location to compute uncorrelated principal components (PCs), linear combinations of the RTF-band values that contain the information present in the original data. The principal components separate the information in the original RTF-band data so that each explains a maximal amount of variance, with most of the information compressed into the first several principal components. Our goal in using PCA was to assess whether the scores of any of the PCs, derived only using RTF-band data, are associated with group (PD vs. control, PDN vs. PDD) membership, and identify the RTF-band-location values with strong contributions to that PC.

We performed two different PCAs. In PCA-A, we used RTF-band data from both PD and healthy control subjects, but not any information on disease status, so that we could subsequently evaluate whether any of the principal components, which only accounted for the variance in the RTF-band data, are associated with membership in the PD or the healthy control cohort. In PCA-B, we used RTF-band data only from PD subjects, but not any information on cognitive status, so that we could subsequently evaluate whether any of the principal components, which only accounted for the variance in the RTF-band data from PD subjects, are associated with cognitive status in PD subjects. Both PCAs used RTF-band values, each centered and scaled to have unit variance, for all four bands at locations C3, C4, O1, O2, F3, F4, F7, F8, FP1, FP2, P3, P4, T3, T4, and T5. Data from midline and ear locations were not included in this analysis, and data for location T6 was unavailable. Logistic regression was used to evaluate the association of a PC’s scores with group membership. The fit of logistic regression models revealing a significant association between a PC and group membership was evaluated as described above; we also assessed whether any observations had a substantial impact on model fit by evaluating the Pearson residuals, deviance residuals and Pregibon leverage. For PD subjects, linear regression was used to evaluate association of a PC with UPDRS-III score or H&Y stage.

To assess uncertainty in the PC scores obtained from the PCAs, we derived their 95% CIs using a bootstrap method with 1,000 replications, using *PCA.Bootstrap* as implemented in *R* (see text footnote 1) by JLR Villardon. This approach overcomes the limitations of asymptotic approaches to calculate CIs (Babamoradi et al., 2013).

## Results

### Relationship of Spectral and Wavelet EEG Analysis

**FIGURE 2:**
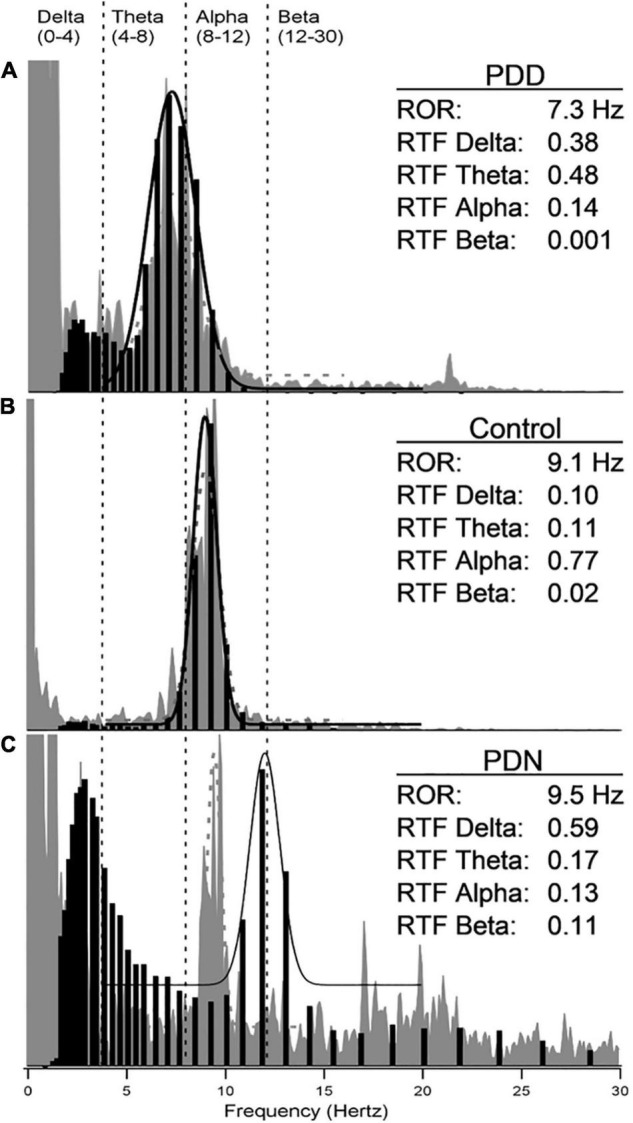
Examples of wavelet-based instantaneous predominant frequency in study subjects. **(A)** (PDD), **(B)** (control), and **(C)** (PDN) show qEEG data obtained at location O1 in the frequency range from 0 to 30 Hz. In gray is the FFT-based resting occipital rhythm (ROR) during 30 s of quiet resting with eyes closed. Black bars show the wavelet-based instantaneous predominant frequency (F0) histogram over the entire recording. A normal curve has been fitted to the FFT (dotted gray) and wavelet-based (black) data over frequencies from 4 to 20 Hz. The ROR and relative times spent by the instantaneous predominant frequency in each EEG band (RTF) are tabulated.

To characterize the relationship of the spectral and wavelet analysis during the 30-min EEG recording in a healthy control, PDN and a PDD individual, the spectral peak representing the background ROR (O1 channel) was calculated by fitting a normal curve to the FFT graph for frequencies of 4–20 Hz. A normal curve was also fit to the histogram of IPF for comparison. Examples of wavelet analysis data in control, PDN and PDD subjects are shown in Figure 2. The IPF histogram and FFT-based spectrogram are often, but not always aligned.

### A Quantitative Electroencephalography Signature Distinguishes Between the Parkinsonian and Control State

#### RTF-Band Values Are Associated With Parkinson’s Disease and Control Status

**FIGURE 3:**
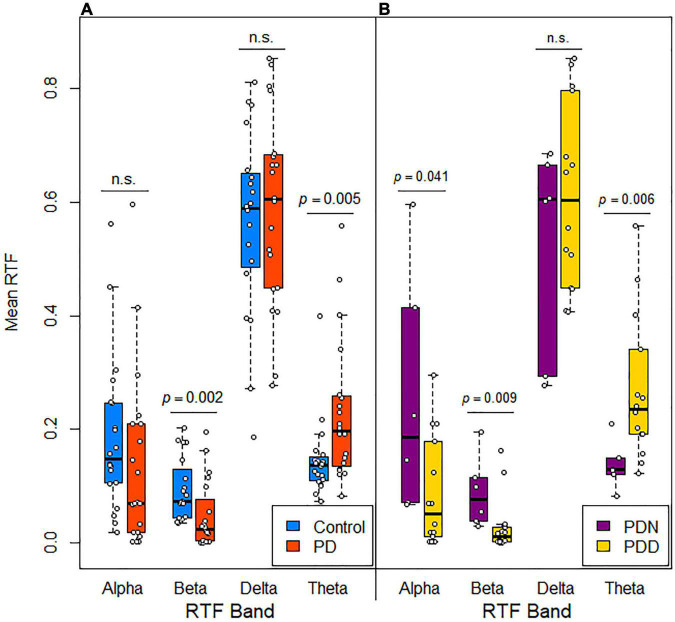
Distribution of mean RTF values in subjects grouped by disease and cognitive status. Box plots with jittered data points show the mean RTF values recorded from all scalp locations for each frequency band in **(A)** control and PD subjects and **(B)** cognitively normal (PDN, MoCA > 25) and cognitively impaired (PDD, MoCA ≤ 25) PD subjects. Significance differences evaluated using a Mann-Whitney *U*-test.

We initially evaluated whether mean and median RTF-band values at all scalp locations were different between control and PD subjects. Compared to control subjects, values in PD subjects were lower for RTF-Beta [TT: control *M* = 0.0930, *SD* = 0.0547; PD *M* = 0.0474, *SD* = 0.0589; *t*(38) = 2.534, *p* = 0.0155; MWU: control *MD* = 0.07, PD *MD* = 0.02, *U* = 3.030, *p* = 0.0020; MT: χ^2^ (1, N = 40) = 6.4, *p* = 0.011], greater for RTF-Theta [TT: control *M* = 0.146, *SD* = 0.069; PD *M* = 0.229, *SD* = 0.125; *t*(38) = –2.599, *p* = 0.0132; MWU: control *MD* = 0.14, PD *MD* = 0.20, *U* = –2.759, *p* = 0.0058; MT: χ^2^ (1, N = 40) = 6.4, *p* = 0.011], and similar for RTF-Delta [TT: control *M* = 0.564, *SD* = 0.163; PD *M* = 0.586, *SD* = 0.171; *t*(38) = –0.403, *p* = 0.689; MWU: control *MD* = 0.59, PD *MD* = 0.60, *U* = –0.541, *p* = 0.6017; MT: χ^2^ (1, N = 40) = 0.4, *p* = 0.527] and RTF-Alpha [TT: control *M* = 0.186, *SD* = 0.135; PD *M* = 0.138, *SD* = 0.155; *t*(38) = 1.048, *p* = 0.301; MWU: control *MD* = 0.15, PD *MD* = 0.07, *U* = 1.542, *p* = 0.1274; MT: χ^2^ (1, N = 40) = 1.6, *p* = 0.206] (Figure 3A).

These results raised the possibility that RTF-band values, possibly in a location-dependent manner, are associated with disease status and degree of cognitive impairment. Therefore, we used logistic regression to explore whether RTF-band values or arithmetic combinations of these values with relatively high variance, which would therefore be more informative for detecting intergroup differences, are associated with disease status. We evaluated arithmetic combinations of RTF-bands that considered the relationships of the faster (alpha and beta) and slower rhythms (delta and theta) in an effort to assess their contribution to the parkinsonian state. A consistent result from these analyses was that RTF-band values in occipital (O1, O2) and parietal (P3, P4) regions were most strongly associated with PD status. The associations were most apparent for arithmetic combinations of RTF-band values [(RTF-Alpha + RTF-Beta)/(RTF-Delta + RTF-Theta), RTF-Alpha + RTF-Beta, RTF-Alpha + RTF-Beta – RTF-Theta, RTF-Alpha – RTF-Theta] and for RTF-Alpha values (Table 1).

**TABLE 1:** Classification of disease status by RTF values at scalp locations using logistic regression.

Scalp locations	RTF or RTF metric evaluated	Variable*	β	95% CI	*P-*value	Area under ROC curve	Classification			McFadden’s adjusted *R*^2^	AIC
							Correct (%)	Sensitivity (%)	Specificity (%)		
O1, O2	$\frac{Alpha+Beta}{Delta+Theta}$	O1	30.25	5.53, 54.96	0.016	0.900	77.5	90.0	65.0	0.308	38.385
		O2	–35.71	–64.92, –6.49	0.017						
	*Alpha + Beta*	O1	45.27	6.83, 83.71	0.021	0.860	75.0	85.0	65.0	0.210	43.804
		O2	–50.58	–90.86, –10.30	0.014						
	*Alpha + Beta – Theta*	O1	23.94	1.48, 46.39	0.037	0.8625	77.5	85.0	70.0	0.210	43.831
		O2	–28.80	–52.87, –4.74	0.019						
P3, P4	*Alpha*	P3	57.11	11.06, 103.15	0.015	0.850	77.5	85.0	70.0	0.278	40.102
		P4	–64.54	–114.17, –14.91	0.011						
	*Alpha – Theta*	P3	23.54	5.76, 41.40	0.010	0.860	80.0	85.0	75.0	0.243	41.957
		P4	–30.99	–52.94, –9.05	0.006						
	*Alpha + Beta – Theta*	P3	12.47	1.172, 23.77	0.031	0.8625	75.0	80.0	70.0	0.170	46.006
		P4	–17.24	–30.30, –4.18	0.010						
	$\frac{Alpha+Beta}{Delta+Theta}$	P3	5.50	0.401, 10.61	0.035	0.8075	67.5	80.0	55.0	0.139	47.76
		P4	–7.823	–14.12, –1.53	0.015						
	*Alpha + Beta*	P3	16.25	0.577, 31.92	0.042	0.8050	70.0	80.0	60.0	0.098	50.098
		P4	–19.71	–36.34, –3.08	0.020						

*The intercept was not significantly different from zero in all models; all models passed the link test.

Significant associations were also seen when RTF-Alpha, RTF-Delta, RTF-Theta, RTF-Alpha-Theta values were evaluated at these scalp locations, when RTF-Beta was evaluated at pairs of T3, T4 and T5, and, consistent with finding that PD subjects have lower mean RTF-Beta and higher mean RTF-Theta, for mean RTF-Theta or mean RTF-Beta. However, the classification, area under the ROC curve, and model-fit statistics were not as strong as those shown in Table 1 (see Supplementary Table 1).

Age and/or sex were not significant predictors when included in any of these analyses. While including sex and/or age often improved classification, they invariably diminished model fit statistics.

#### Principal Component Analysis Identifies a Principal Component Able to Distinguish Between Parkinson’s Disease and Control Subjects

Since RTF values within a frequency band, and arithmetic combinations of RTF values of different frequency bands tend to be correlated (Table 2), we were concerned that co-linearity among variables diluted the precision of the logistic regression analyses. Therefore, we used PCA to reduce data dimensionality and assess whether uncorrelated PCs are associated with PD vs. control status. PCA-A was performed using the only the RTF-band values in each frequency band, from all subjects, at scalp locations C3, C4, O1, O2, F3, F4, F7, F8, FP1, FP2, P3, P4, T3, T4, and T5.

**TABLE 2:** Correlation between RTF values across all scalp locations.

Band	Mean (Range)
Alpha	0.97 (0.92–0.99)
Beta	0.69 (0.071–0.99)
Delta	0.90 (0.59–0.99)
Theta	0.85 (0.56–0.99)
Alpha – Beta	0.86 (0.37–0.99)
Alpha + Beta – Theta	0.81 (0.22–0.99)
Alpha – Theta	0.90 (0.72–0.99)
Theta – Beta	0.74 (0.034–0.99)
(Alpha + Beta) (Delta + Theta)	0.82 (0.16–0.99)

**FIGURE 4:**
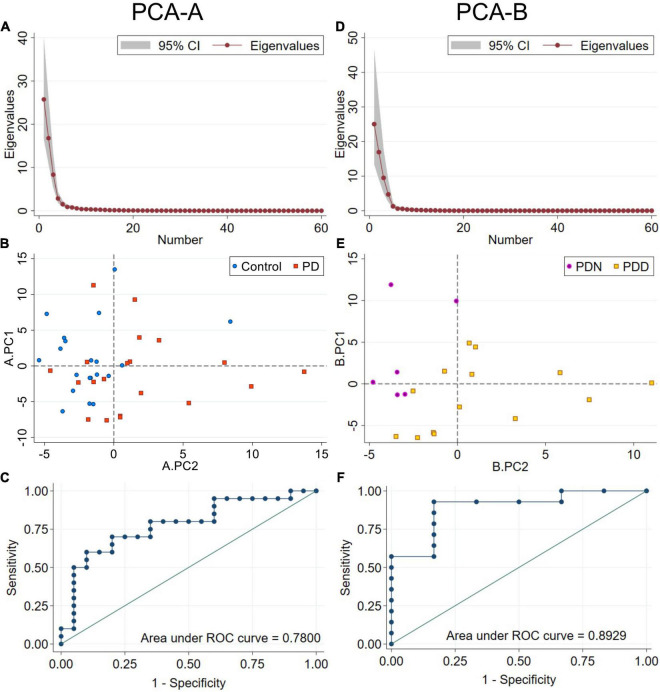
PCA using RTF data for each frequency band at all scalp locations. PCA-A used RTF data from PD and control subjects. **(A)** Scree plot of eigenvalues for PCA-A. Principal components A.PC1–5 account for 92.0% of the total variance. **(B)** Distribution of control (blue circles) and PD (orange squares) subjects by A.PC1 and A.PC2 scores. A.PC2 scores distribute subjects by disease status more effectively than A.PC1 scores. **(C)** ROC curve from a logistic regression model classifying subjects by disease status using A.PC2 scores. PCA-B used RTF data only from PD subjects. **(D)** Scree plot of eigenvalues for PCA-B. Principal components B.PC1–5 account for 95.8% of the total variance. **(E)** Distribution of PDN (MoCA ≥ 26, purple circles) and PDD (MoCA ≤ 25, gold squares) subjects by B.PC1 and B.PC2 scores. B.PC2 scores distribute PDN from PDD subjects more effectively than B.PC1 scores. **(F)** ROC curve from a logistic regression model classifying PD subjects by PDN vs. PDD status using B.PC2 scores.

The first five PCs (named here A.PC1 – A.PC5 to avoid confusion with PCs generated in the second PCA-B described below), which explain 91.96% of the total variance (Figure 4A) (A.PC1, 42.9%; A.PC2, 27.9%; A.PC3, 13.9%; A.PC4, 4.72%; A.PC5, 2.5%) were each evaluated for a possible association with PD or control status using logistic regression. We found that A.PC2 is associated with PD and control status (model *p* = 0.0037; β = 0.31, 95% CI: 0.037, 0.59; A.PC2 *p* value: 0.027; Intercept not significantly different from zero; McFadden’s adjusted *R*^2^ = 0.280; Goodness-of-fit-test Pearson’s χ^2^ = 46.85, *p* = 0.154; Link test: pass; AIC = 51.02; Area under ROC curve (AUC): 0.780; Classification: 70% correct, 60% sensitivity, 80% specificity) (Figures 4B,C). While A.PC2 remained a significant predictor of disease status in models incorporating sex and age, age and sex, either individually or together, were not significant predictors of disease status. Including them did not improve model fit as evaluated using AIC or McFadden’s Adjusted *R*^2^, though including both improved classification (correct: 80.0%; sensitivity: 75%; specificity: 85%; AUC = 0.8225). A.PC1, A.PC3, A.PC4, and A.PC5 were not associated with PD vs. control status (model *p* = 0.231, 0.453, 0.695, 0.355, respectively). A bootstrap analysis deriving the 95% CIs for the A.PC1 and A.PC2 scores for each study subject, as well as other PCA parameters, indicated that the PCA results are stable and reproducible (Supplementary Figure 1).

**FIGURE 5:**
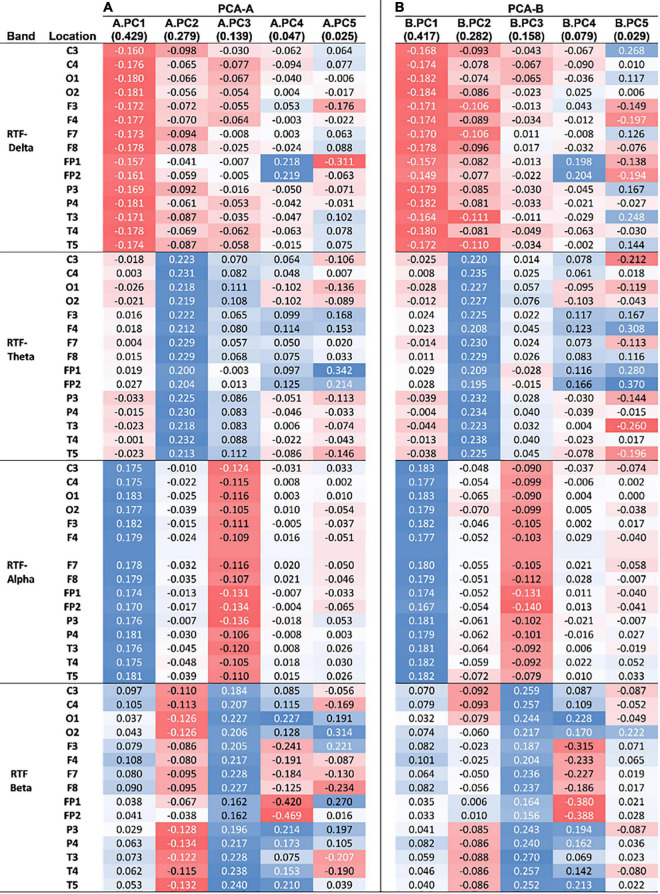
Loadings of uncorrelated principal components from PCAs. **(A)** Loadings from the first five PCs of PCA-A (A.PC1–5), which used RTF values from scalp locations in all subjects. **(B)** Loadings from the first five PCs of PCA-B (B.PC1–5), which used RTF values only from PD subjects. The amount of variance explained by each PC is indicated parenthetically. The intensity and color of shading reflects the strength (stronger = more intense) and type of correlation (red = negative, blue = positive) of the loading with a PC.

Examination of the PCA-A loadings revealed that the loadings for A.PC2 have strong, positive and similar levels of contribution from RTF-Theta values at all scalp locations, and strong negative contributions from RTF-Beta values at locations O1, O2, P3, P4, C3, C4, T4, T5, and T6 (Figure 5A). These results are congruent with those found in our initial analyses: (1) Compared to control subjects, PD subjects had lower average RTF-Beta and greater average RTF-Theta, and (2) Arithmetic combinations of RTF-band values that include the beta and theta bands at occipital, as well as parietal locations, have utility in discriminating between PD and control subjects.

### A Quantitative Electroencephalography Signature Is Associated With Cognitive Status in the Parkinsonian State

We took a similar approach to that used to identify qEEG features distinguishing controls from PD subjects to evaluate whether a qEEG signature is associated with cognitive status in the parkinsonian state. First, we compared the means of the RTF-band values at all scalp locations in PDN (cognitively normal) and PDD (cognitively impaired subjects). Compared to PDN subjects, median values in PDD subjects were lower for RTF-Alpha [MWU: PDN *MD* = 0.19, PDD *MD* = 0.05, *U* = 2.062, *p* = 0.0408; MT: χ^2^ (1, N = 20) = 3.81, *p* = 0.05], lower for RTF-Beta [MWU: PDN *MD* = 0.08, PDD *MD* = 0.01, *U* = 2.557, *p* = 0.0087; MT: χ^2^ (1, N = 20) = 8.57, *p* = 0.003], greater for RTF-Theta [MWU: PDN *MD* = 0.13, PDD *MD* = 0.24, *U* = –2.638, *p* = 0.0064; MT: χ^2^ (1, N = 20) = 3.81, *p* = 0.05] and similar for RTF-Delta [MWU: PDN *MD* = 0.60, PDD *MD* = 0.60, *U* = –0.825, *p* = 0.4095; MT: χ^2^ (1, N = 20) = 0.0, *p* = 1.0] (Figure 3B). This suggested that RTF-band values at some locations, or the average RTF-band value across all scalp locations, might be useful to discriminate between PDN and PDD subjects.

To explore this hypothesis, we used logistic regression in an attempt to identify RTF locations and frequency bands associated with cognitive status in the parkinsonian state. While some RTF-band variables (RTF-Theta in C3 and C4, RTF-Theta in O1 and O2, RTF-Theta in P3 and P4; RTF-Beta in C3 and C4, RTF-Beta in T3, T4, or T5) resulted in models with a model *p* < 0.05, either RTF-band variables did contribute significantly to the models, or a link test run after the estimation commands failed, suggesting that none of the models were properly specified (Supplementary Table 2).

We therefore evaluated whether PCA could identify uncorrelated PCs useful for classification of PD subjects by their cognitive status (PDN vs. PDD). PCA-B was done using RTF-band values = at the same scalp locations used in PCA-A, but only from PD subjects. The first five PCs (named here B.PC1 – B.PC5 to distinguish them from A.PC1-5 described above) accounted for 95.75 percent of the total variance (B.PC1, 41.73%; B.PC2, 28.23%; B.PC3, 15.83%; B.PC4, 7.86%; B.PC5, 2.89%) (Figure 4D). We used logistic regression to evaluate whether any of the first five B.PCs were associated with PDN vs. PDD status. While neither age (*p* = 0.634), disease duration (*p* = 0.444), nor sex (*p* = 0.116) were significantly associated with cognitive status (Table 3), since disease duration and age are risk factors for cognitive impairment, we evaluated whether including these covariates improved model-fit statistics. We found that B.PC2 was associated with PDN v PDD status (Figures 4E,F), and that including age, disease duration and/or sex as covariates did not improve model fit statistics (Table 3). While some models evaluating the association of B.PC1 and the covariates with PDN/PDD status were significant, their model-fit statistics suggested they were not properly specified. Models evaluating the association of B.PC3, B.PC4, and B.PC5 with PDN/PDD status were not significant (model *p* = 0.421, 0.357, 0.326, respectively). As for PCA-A, a bootstrap analysis deriving the 95% CIs associated with B.PC1 and B.PC2 scores, as well as other PCA parameters, indicated that the PCA results are stable and reproducible (Supplementary Figure 2).

**TABLE 3:** Results of logistic regression-based classification of cognitive status in the parkinsonian state by B.PC1, B.PC2, disease duration, age and sex.

Independent variables	Model P	Variable*	β	95% CI	*P-*value	Area under ROC curve	Classification			McFadden’s adjusted *R*^2^	AIC	Link test
							Correct (%)	Sensitivity (%)	Specificity (%)			
Disease duration	0.444	Duration	0.134	–0.239, 0.507	0.401	0.5179	70.0	100.0	0.0	–0.140	27.849	Fail
Age	0.634	Age	0.029	–0.093, 0.153	0.637	0.5595	70.0	100.0	0.0	–0.154	28.208	Fail
Sex	0.112	Sex	–1.609	–3.66, 0.446	0.125	0.690	70.0	100.0	0.0	–0.060	25.903	Fail
PC1	0.0340	PC1	–0.236	–0.494, –0.0213	0.072	0.7381	70.0	85.71	33.33	0.020	23.942	Fail
B.PC1 + Disease Duration	0.0945	PC1	–0.231	–0.491, 0.039	0.082	0.7500	70.0	85.71	33.33	–0.052	25.716	Fail
		Duration	0.084	–0.280, 0.449	0.652							
B.PC1 + Age	0.0242	PC1	–0.369	–0.711, –0.026	0.035	0.8571	85.0	92.86	66.67	0.059	22.994	Fail
		Age	0.155	–0.048, 0.358	0.134							
B.PC1 + Sex	0.0282	PC1	–0.297	–0.646, –0.052	0.095	0.8333	70.0	85.71	33.33	0.047	23.298	Fail
		Sex	–1.971	–4.594, 0.653	0.141							
B.PC1 + Disease Duration + Age + Sex*	0.0095	PC1	–0.643	–1.302, 0.0376	0.064	0.9405	85.0	92.86	66.67	0.139	21.04	Fail
		Duration	–0.395	–1.303, 0.512	0.393							
		Age	0.389	–0.177, 0.955	0.178							
		Sex	–5.00	–12.29, 2.29	0.179							
B.PC2	0.0020	PC2	0.922	0.019, 1.925	0.045	0.8929	90.0	92.86	83.33	0.226	18.921	Pass
B.PC2 + Disease Duration	0.0085	PC2	0.985	–0.228, 1.833	0.056	0.8929	90.0	92.86	83.33	0.144	20.984	Pass
PC2 + Age	0.0041	PC2	1.295	0.0151, 2.575	0.047	0.9048	90.0	85.7	66.67	0.205	19.417	Pass
		Age	–0.1424	–0.406, 0.121	0.289							
PC2 + Sex	0.0067	PC2	0.813	–0.100, 1.734	0.084	0.9048	85.0	85.71	83.3	0.164	20.425	Pass
		Sex	–0.957	–3.611, 1.696	0.479							
PC2 + Disease Duration + Age + Sex	0.0250	PC2	1.270	–0.224, 2,765	0.096	0.9048	90.0	92.86	83.33	0.044	23.36	Fail
		Duration	–0.051	–0.588, 0.475	0.850							
		Age	–0.132	–0.420, 0.156	0.368							
		Sex	–0.392	–3.98, 3.19	0.830							

*In all models, the intercept was not significantly different from zero.

Examination of the PCA-B loadings revealed that that B.PC2 has positive, similar strong level of contribution from RTF-Theta values at all scalp locations. Less strong are negative contributions from RTF-Beta values at central locations (C3, C4) and RTF-Delta levels at central (C3), frontal (F3, F7, F8), and temporal (T3, T4) locations (Figure 5B). Compared to the PCA-A loadings distinguishing between the PD and normal state, the PCA-B loadings distinguishing between normal and impaired cognition in PD that involve RTF-Beta are generally less strong while those involving RTF-Delta are generally stronger.

To address whether qEEG features associated with PDN vs. PDD status might also be associated with the motor or disease state, we used linear regression to evaluate if B.PC1 – B.PC5 scores were associated with scores on UPDRS-III or with H&Y stage. These analyses did not reveal any significant associations (UPDRS-III: B.PC1, *p* = 0.498, *R*^2^ = 0.0259; B.PC2, *p* = 0.587, *R*^2^ = 0.0167; B.PC3, *p* = 0.233, *R*^2^ = 0.0267; B.PC4, *p* = 0.536, *R*^2^ = 0.0216; B.PC5, *p* = 0.449, *R*^2^ = 0.322. H&Y stage: B.PC1, *p* = 0.186, *R*^2^ = 0.168; B.PC2, *p* = 0.950, *R*^2^ = 0.0004; B.PC3, *p* = 0.961, *R*^2^ = 0.0002; B.PC4, *p* = 0.722, *R*^2^ = 0.0132; B.PC5, *p* = 0.282, *R*^2^ = 0.115).

## Discussion

Here we present a retrospective, cross-sectional analysis of standard EEG recordings in a cohort of PD patients and healthy controls from a clinical practice setting. We demonstrate that the novel application of a wavelet-based transform to these routinely gathered clinical data identifies a qEEG signature that may assist in predicting cognitive dysfunction in PD patients. The EEG data can contain one or more independent spectral components that may exhibit rapid variation in their frequency based on the underlying cortical dynamics. The wavelet transform method can capture these rapid changes in frequency and can help derive the instantaneous frequency of the EEG signal at multiple time scales (Indic and Narayanan, 2011). As such, the wavelet-based analysis used in our study has the advantage of more accurately reflecting the dynamic character of non-stationary EEG signal at any given time, in comparison to the Fourier or Hilbert transform (Wacker and Witte, 2013).

Our results are in full agreement with previous reports indicating that qEEG has utility in distinguishing the parkinsonian state from healthy controls, as well as characterizing the presence of cognitive impairment. In a review of 23 studies assessing cognition in PD, Geraedts et al. (2018) reported that in cross-sectional studies, EEG slowing correlated with cognitive impairment, whereas in longitudinal studies, decreased dominant frequency and increased theta power correlated with cognitive impairment and predicted future cognitive deterioration.

Logistic regression showed that arithmetic combinations of RTF-band values at occipital and parietal locations such as (RTF-Alpha + RTF-Beta)/(RTF-Delta + RTF-Theta) can distinguish between the PD and control states. Furthermore, PCA using RTF-band values from both control and PD subjects identified a PC with contributions from RTF-Theta and RTF-Beta at all scalp locations that can distinguish between PD and control state with an AUC of 0.78. A separate PCA using RTF-band values only from PD subjects identified a PC able to distinguish between PDN and PDD with an AUC of 0.8929. Compared to the PC associated with PD vs. control status, the PC associated with PDN vs. PDD cognitive status had differing contributions from RTF-Delta and RTF-Beta originating in the frontal and occipital regions.

Our analysis highlights the contribution of different EEG dynamics to the parkinsonian vs. non-disease state and cognitive impairment within the parkinsonian state. Comparing the loadings of the PCs associated with each of these dichotomous states reveals positive associations of RTF-Theta and negative associations of RTF-Delta and RTF-Beta in both the parkinsonian state and cognitive impairment within the parkinsonian state, however, relative to the associations distinguishing the parkinsonian and control state, the negative associations with cognitive impairment in parkinsonian subjects are relatively stronger for RTF-Delta and relatively weaker for RTF-Beta. More specifically, the associations of cognitive impairment with RTF-values in frontal, temporal and occipital locations appear to be relatively stronger for RTF-Delta and relatively weaker for RTF-Beta (Figure 5). These findings suggest that the EEG state associated with disease status is not identical to that of cognitive impairment. This difference appears to be specific to the cognitive state as there is no association to the severity of the motor state or disease stage.

Beta oscillations in the basal ganglia have been associated with an anti-kinetic motor state in PD (Little and Brown, 2014). Sustained synchronization between the subthalamic nucleus (STN) and premotor-cortical oscillations likely disrupts normal movement in PD (Sharott et al., 2018). Furthermore, reduction of basal-ganglia beta oscillations by STN deep-brain stimulation (STN-DBS) has been associated with symptomatic improvement (Yin et al., 2021). It is conceivable that the decreased RTF-Beta we observe reflects impaired synchrony between the basal ganglia and the cortex. Cole et al. (2017) showed that non-sinusoidal beta oscillations may underlie cortical pathophysiology in PD and may reflect input synchrony onto the cortex. In that context, it is conceivable that Fourier-based methods fail to fully capture the temporal localization of the frequencies due to the non-stationary nature of EEG data, and so represent the frequencies as present throughout the recording duration. Similarly, the amplitude corresponding to the represented frequencies was depicted as being smooth and present throughout the recording duration. While our study was not designed to assess the role of cortical beta oscillations, the wavelet transform that we used can capture rapid and discrete changes in frequency and as such may more accurately capture the underlying cortical functional state.

Our study has several limitations: One is its retrospective cross-sectional design and a second is its modest sample size. Longitudinal follow-up of the non-demented patients with assessment of the qEEG variables and cognitive status at annual intervals could help confirm the findings of this pilot study. Our measures of effect size will be useful to evaluate sample-size requirements for a larger prospective study. A third limitation is the lack of detailed characterization of the cognitive status of the controls. The controls were selected from the electronic medical record (EMR) and were based on the following criteria: normal EEG, a diagnosis of syncope and absence of a diagnosis of neurological disorder or cognitive impairment. These controls were not clinically assessed and therefore, subtle symptoms associated with later onset of a neurological disease may have been missed.

A fourth limitation is that the PD subjects were on dopaminergic medications when EEGs were recorded. The potential contribution of dopaminergic therapy on brain activity and consequently on the EEG in either the parkinsonian or healthy state cannot be excluded. Dopamine has been shown to modulate cortico-subthalamic activity in in the parkinsonian state and dopaminergic medications lead to coherence in the delta/theta range in the medial and orbitofrontal cortex (Sharma et al., 2021). Furthermore, dopamine can have an effect in the healthy brain by modulating brain dynamics (Braun et al., 2021). Beta oscillations are associated with motor symptoms in PD (van Wijk et al., 2016). Dopaminergic medication can induce changes in phase-amplitude coupling and distributed coherence, which are correlated with changes in rigidity in PD subjects (Miller et al., 2019), so such medication impacts the elevated synchronization seen in PD. Also, dopaminergic medications can be associated with cognitive impairment that may be both brain-region-specific (MacDonald et al., 2011), and dose-dependent (Macdonald and Monchi, 2011). The potential for multifaceted actions of dopamine in the healthy and parkinsonian state does not allow for a clear prediction of how dopaminergic medications would impact RTF-band values in a qEEG analysis. Therefore, the extent to which dopaminergic therapy impacts our findings with respect to cognition warrants further study.

In conclusion, the findings of this retrospective study provide support for the utility of using the novel application of a wavelet-based transform qEEG method to distinguish the parkinsonian state and identify qEEG parameters that can serve as a biomarker for the declining cognitive status in PD patients. Longitudinal assessment of these qEEG parameters and correlation with detailed clinical phenotyping as well as CSF and imaging biomarkers in a larger cohort will provide further insight into PD progression, the progression of cognitive impairment within PD, and the underlying neurodegenerative process.

## Data Availability Statement

The datasets presented in this article are not readily available because the data contain protected health information (PHI). The raw data without PHI can be made available on request from the corresponding author. Requests to access the datasets should be directed to corresponding author KM, amarkopoulou@northshore.org.

## Ethics Statement

This was a retrospective study analyzing data obtained as part of standard clinical practice. The studies involving human participants were reviewed and approved by NorthShore University HealthSystem Institutional Review Board. Written informed consent from the patients/participants was not required in this study in accordance with the national legislation and the institutional requirements.

## Author Contributions

KM, JN, and KN designed the study. PI performed the wavelet analysis. KN and BC performed data and statistical analysis. All authors participated in editing the manuscript and approved its content.

## Conflict of Interest

The authors declare that the research was conducted in the absence of any commercial or financial relationships that could be construed as a potential conflict of interest.

## Publisher’s Note

All claims expressed in this article are solely those of the authors and do not necessarily represent those of their affiliated organizations, or those of the publisher, the editors and the reviewers. Any product that may be evaluated in this article, or claim that may be made by its manufacturer, is not guaranteed or endorsed by the publisher.

## Footnotes

1
https://CRAN.R-project.org

## Abbreviations

AIC: Aikake information criterion
AUC: Area under the receiver operating characteristic curve
CI: Confidence interval
EEG: Electroencephalogram
EMR: Electronic medical record
FFT: Fast Fourier transform
H&Y: stage Hoehn and Yahr Parkinson’s disease stage
IPF: The instantaneous predominant frequency at a scalp location, calculated at 60 ms intervals throughout a resting-state EEG recording
LB: Lewy bodies
MCI: Mild cognitive impairment
MCI-AD: Mild cognitive impairment associated with Alzheimer’s disease
MCI-LB: Mild cognitive impairment associated with Lewy bodies
MD: Median
MoCA: Montreal Cognitive Assessment
MT: Median test
MWU: Mann-Whitney *U*-test
PC: Principal component
PCA: Principal components analysis
PD: Parkinson’s disease
PDD: Parkinson’s disease subjects with impaired cognition (MoCA ≤ 25)
PDN: Parkinson’s disease subjects with normal cognition (MoCA 26-30)
qEEG: Quantitative electroencephalography
ROR: Resting occipital rhythm
RTF-band: The relative time spent by the IPF in one of the four standard EEG frequency bands (alpha, beta, delta, or theta) during an entire EEG recording
SD: Standard deviation
STN: Subthalamic nucleus
STN-DBS: Subthalamic nucleus deep brain stimulation
TT: *t*-test (two-tailed)
UPDRS-III: Unified Parkinson’s Disease Rating Scale, part III (motor score).
