# Supplementary material for: Quantitative Electroencephalography as a Biomarker for Cognitive Dysfunction in Parkinson’s Disease
Source: Front Aging Neurosci. 2022 Jan 3;13:804991. doi: 10.3389/fnagi.2021.804991 (PMC8761986; doi:10.3389/fnagi.2021.804991)

Supplemental Table 1. Additional data on classification of disease status by RTF-band values at scalp locations using logistic regression.

Scalp Locations	RTF or RTF Metric Evaluated	Model *P* Value	Variable*	β	95% CI	*P* Value	Area under ROC curve	Classification			McFadden's Adjusted R^2	AIC	Link Test
								Correct (%)	Sensitivity (%)	Specificity (%)			
O1, O2	*Alpha*	0.0097	O1	31.06	0.48, 61.65	0.047	0.758	67.5	80.0	55.0	0.059	52.18	Pass
			O2	–34.71	–67.01, –2.41	0.035							
	Delta	0.0138	O1	–25.82	–50.66, –0.99	0.042	0.720	65.0	70.0	60.0	0.046	52.83	Pass
			O2	27.28	1.95, 52.62	0.035							
	Theta	0.0074	O1	–15.79	–48.01, 16.42	0.337	0.775	70.0	60.0	80.0	0.069	51.64	Pass
			O2	25.24	–8.89, 59.37	0.147							
			Intercept	–1.93	–3.53, –0.34	0.018							
	Alpha – Theta	0.0034	O1	18.52	–2.64, 39.67	0.086	0.800	75.0	80.0	70.0	0.096	50.11	Pass
			O2	–22.80	–45.61, 0.01	0.050							
P3, P4	*Alpha*	0.0000	P3	57.11	11.06, 103.15	0.015	0.850	77.5	85.0	70.0	0.278	40.04	Pass
			P4	–64.54	–114.2, –14.91	0.011							
	Delta	0.0410	P3	–12.80	–25.42, –0.17	0.047	0.688	62.5	70.0	55.0	0.007	55.06	Pass
			P4	12.90	0.434, 25,37	0.043							
	Theta	0.0065	P3	–6.28	–25.54, 12.97	0.522	0.800	70.0	55.0	85.0	0.074	51.36	Pass
			P4	17.81	–4,89, 40.52	0.124							
			Intercept	–2.04	–3.73, –0.35	0.018							
	Alpha – Theta	0.0001	P3	23.54	5.67, 41.40	0.010	0.860	80.0	85.0	75.0	0.243	41.96	Pass
			P4	–31.00	–52.95, –9.05	0.006							
T3. T4		0.0012	T3	–54.80	–94.88, –14.72	0.007	0.818	72.5	75.0	70.0	0.134	48.00	Pass

	Beta		T4	31.21	0.93, 61.48	0.043						
			Intercept	1.25	0.834, 2.42	0.036						
T3, T5	*Beta*	0.0199	T3	–17.17	–41,51, 7.16	0.167	0.775	67.5	75.0	60.0	0.033	52.68	Pass
			T5	–0.335	–23.02, 22.35	0.977						
			Intercept	1.11	0.035, 2.189	0.043						
T4, T5	*Beta*	0.0264	T4	14.62	–10.51, 39.77	0.254	0.748	72.50	80.0	65.0	0.023	54.18	Pass
			T5	–29.46	–59.80, 0.878	0.059						
Mean RTF-*Theta*		0.0129	Mean RTF-*Theta*	–14.56	–27.29, –1.83	0.025	0.780	72.50	75.0	70.0	0.039	53.27	Fail
Mean RTF-*Beta*		0.0070	Mean RTF-*Beta*	10.85	0.645, 21.06	0.037	0.755	72.50	60.0	85.0	0.059	52.19	Pass
			Intercept	–1.91	–3.70, –0.115	0.037						

*The intercept was not significantly different from zero except where indicated.

Supplemental Table 2. Additional data on classification of cognitive status in PD subjects by RTF-band values at scalp locations using logistic regression.

Scalp Locations	RTF or RTF Metric Evaluated	Model *P* Value	Variable*	β	95% CI	*P* Value	Area under ROC curve	Classification: Correct (%)	Classification: Sensitivity (%)	Classification: Specificity (%)	McFadden's Adjusted R^2	AIC	Link Test
O1, O2	*Theta*	0.008	O1	87.89	–8.46, 184.23	0.074	0.952	90.0	92.9	83.3	0.340	16.13	Fail
			O2	–44.06	–116.02, 27.89	0.230							
			Intercept	–6.07	–13.32, 1.18	0.101							
C3, C4	*Theta*	0.0062	C3	49.94	–9.93, 109.81	0.102	0.881	80.0	85,7	66.7	0.170	29.28	Fail
			C4	–20.51	–62.92, 21.89	0.343							
			Intercept	–4.05	–8.82, 0.72	0.096							
	Beta	0.0120	C3	–36.22	–101.80, 29.36	0.279	0.905	80.0	85.7	66.7	0.116	21.59	Pass
			C4	7.14	–39.10, 55.39	0.762							
			Intercept	2.67	0.55, 4.79	0.014							
P3, P4	*Theta*	0.0008	P3	74.07	–14.12, 162.25	0.100	0.940	90.0	92.9	83.3	0.335	16.24	Fail
			P4	–34.01	–97.07, 29.05	0.029							
			Intercept	–4.67	–10.31, –0.98	0.105							
T3	*Beta*	0.0204	T3	–25.31	–50.38, –0.25	0.048	0.845	70.0	85.7	33.3	0.056	23.06	Fail
			Intercept	2.07	0.40, 3.75	0.015							
T4	*Beta*	0.0250	T4	–17.52	–37.02, 1.98	0.078	0.821	70.0	85.7	33.3	0.042	23.41	Fail
			Intercept	1.91	0.33, 3.49	0.018							
T5	*Beta*	0.0305	T5	–21.38	–46.77, 4.00	0.099	0.881	75.0	92.9	33.3	0.028	23.75	Fail
			Intercept	1.61	0.28, 2.94	0.018							

Supplemental Figure 1. 95% confidence intervals for A.PC1 and A.PC2. 95% CIs for PCA-A parameters were calculated using a bootstrap method with 1,000 replications. The graph plots the scores of study-subjects for A.PC1 and A.PC2. Healthy-controls are represented by orange rectangles and PD subjects by blue rectangles, with each rectangle centered over the point defined by their A.PC1 and A.PC2 scores. The width of each rectangle corresponds to the 95% CI of a subjects A.PC2 score. The height of each rectangle corresponds to the 95% CI of a subjects A.PC1 score. Compare to Figure 4B.

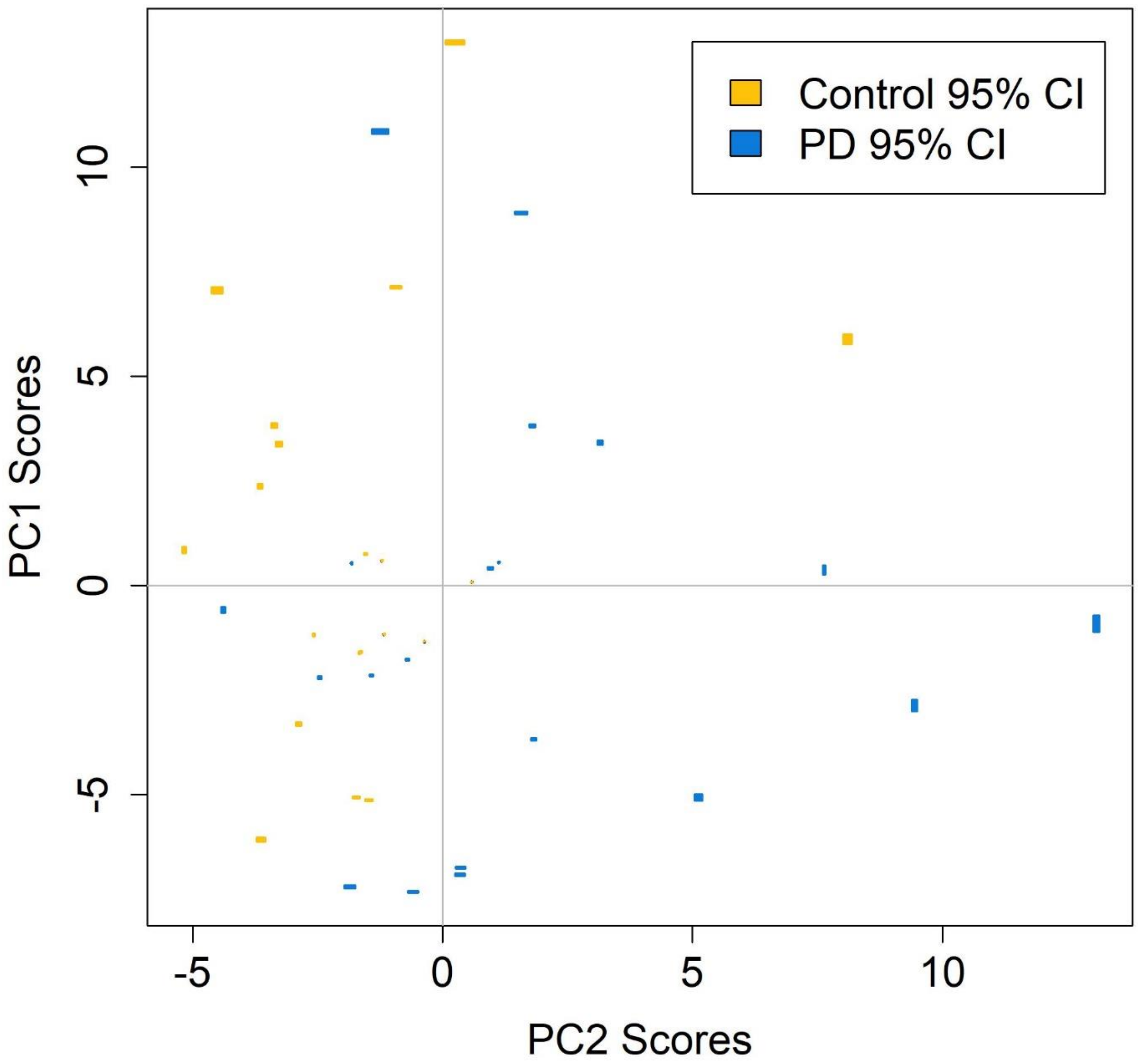

Supplemental Figure 2. 95% confidence intervals for B.PC1 and B.PC2. 95% CIs for PCA-B parameters were calculated using a bootstrap method with 1,000 replications. The graph plots the scores of study-subjects for B.PC1 and B.PC2. PD Subjects with cognitive impairment (PDD, MoCA ≤25) are represented by gold rectangles and PD subjects with normal cognition (PDN, MoCA 26-30) are represented by blue rectangles, with each rectangle centered over the point defined by their B.PC1 and B.PC2 scores. The width of each rectangle corresponds to the 95% CI of a subjects B.PC2 score. The height of each rectangle corresponds to the 95% CI of a subjects B.PC1 score. Compare to Figure 4E.

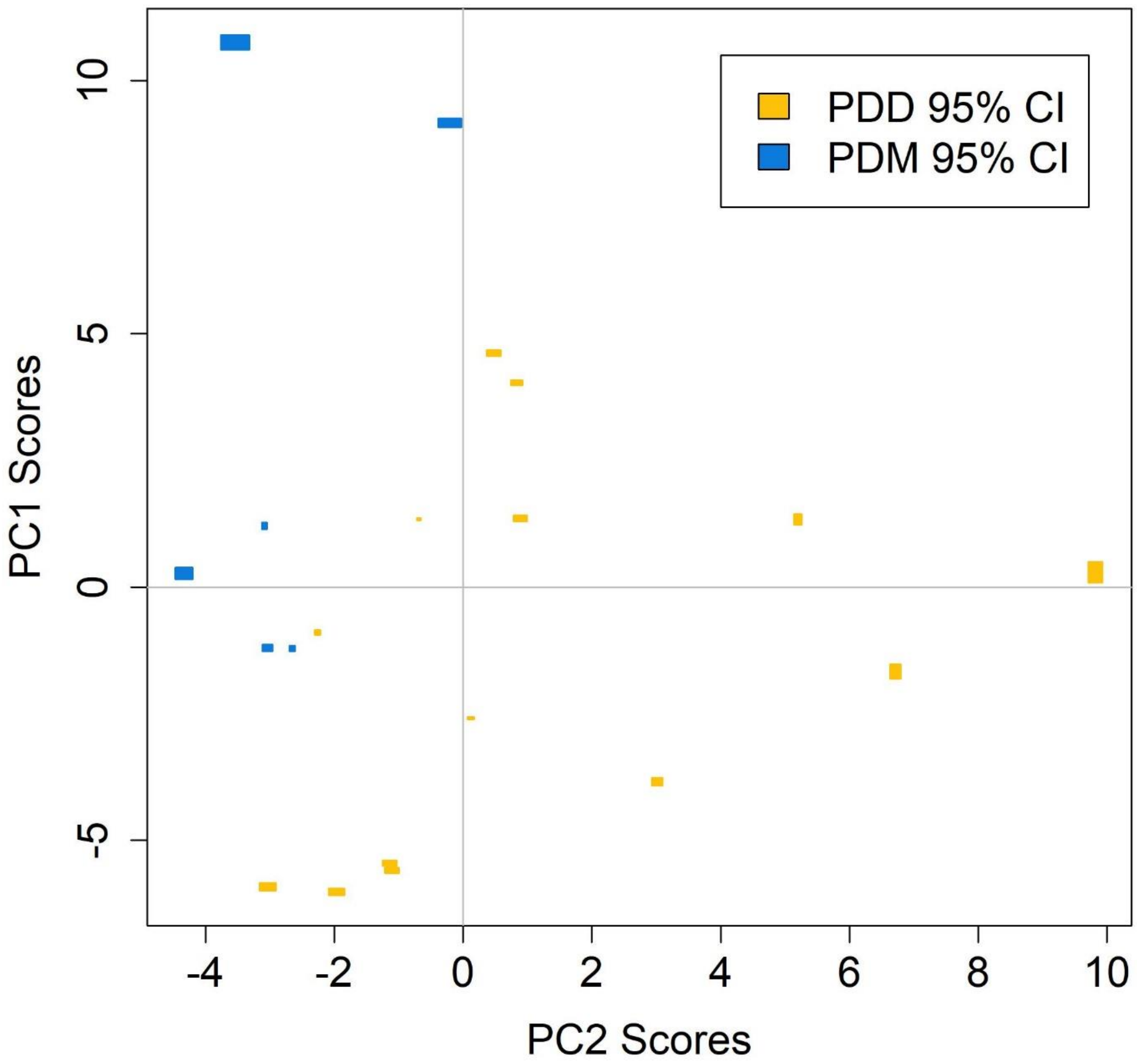

Supplement: Supplementary file 1 [file Data_Sheet_1.PDF]
